# IRT analysis of the BDI-II for early online depression detection: validation in a Mexican population

**DOI:** 10.3389/fpsyg.2025.1562016

**Published:** 2025-04-15

**Authors:** Tonathiu Salcedo-Callado, Norberto Hernández-Llanes, Ricardo Sánchez-Domínguez, Ricardo Saracco-Alvarez, Rodrigo Marín-Navarrete

**Affiliations:** ^1^Departamento de Investigación Psicosocial y Documental, Centros de Integración Juvenil, A.C., Mexico City, Mexico; ^2^Subdirección de Investigaciones Clínicas, Instituto Nacional de Psiquiatría, Mexico City, Mexico; ^3^Dirección de Investigación y Enseñanza, Centros de Integración Juvenil, A.C., Mexico City, Mexico

**Keywords:** validation study, affective disorders, self-assessments, health services, eHealth

## Abstract

**Introduction:**

Identifying factors associated with depression is crucial to addressing the global rise in mental health needs. The Beck Depression Inventory II (BDI-II) has shown robustness in assessing depression, even in digital contexts. However, psychometric evidence is essential to support its use in online self-diagnosis, particularly in regions where it has not been widely employed for this purpose.

**Objective:**

This study aimed to evaluate the psychometric properties of the BDI-II for online self-diagnosis among Mexican adults.

**Method:**

Data from 58,456 medical records were analysed using Item Response Theory (IRT).

**Results:**

A good fit was found for a hierarchical confirmatory model with 1 s-order factor (overall severity) and two first-order factors (cognitive and somatic symptoms), as well as optimal accuracy estimates in both the IRT and the Classical Test Theory (CTT).

**Discussion:**

These findings support the use of the BDI-II as a reliable online screening tool for depression in self-diagnosis settings for Mexican adults.

## 1 Introduction

The escalating demand for mental health services over the past decade has resulted in a care deficit (Kim et al., 2023), necessitating the implementation of innovative strategies and tools for the early identification of mental disorders and timely referral to treatment. Technical and technological advancements have facilitated the development of e-Health tools, which are digital or online tools focused on healthcare (Jacob et al., 2023). When these tools specifically address mental health, they are designated as e-mHealth tools. Their application in public health services has demonstrated the potential to alleviate service demand and enhance healthcare accessibility for populations historically marginalized due to mobility limitations or disabilities (Freeman, 2022; Jacobson et al., 2022; Jonsson et al., 2023; Sin et al., 2020; Spanhel et al., 2021).

There are e-mHealth tools designed for the self-detection of mental health conditions are increasingly prevalent (Jacobson et al., 2022; Dieris-Hirche et al., 2023; Fischer et al., 2025; Whitton et al., 2021; Zarp et al., 2025). The use of these tools for self-assessment in mental health has been acknowledged as a potentially valuable approach for the early identification of mental disorders. Nonetheless, their accuracy and validity for both users and healthcare providers necessitate rigorous evaluation (Funnell et al., 2024). Some tools have demonstrated effectiveness in identifying the risk of commonly diagnosed and increasingly prevalent disorders, such as depression (Esser et al., 2018; Park et al., 2020; Parker et al., 2020; Wang et al., 2018).

In this regard, depression is the most prevalent of all mental disorders. In 2019, there were an estimated 3,440.1 cases per 100,000 inhabitants worldwide, accounting for 28.1% of all those with a mental illness (Global Burden of Disease Collaborative Network, 2022). In Mexico, its prevalence rose to 31.1% in adolescents and 16.7% in adults in 2022 (Vázquez-Salas et al., 2022).

Consequently, numerous efforts have been made to develop tools that permit the timely detection of depression in a range of settings and populations. Examples of the scales used include The Montgomery-Åsberg Depression Rating Scale—Self-rated [MADRS-S] (Montgomery and Åsberg, 1979), the Patient Health Questionnaire-9 [PHQ-9] (Kroenke et al., 2001), the Hospital and Depression Scale [HADS-A] (Zigmond and Snaith, 1983) and the Beck Depression Inventory [BDI-II] (Beck et al., 1996). The latter is one of the most commonly used scales for measuring depression and has been adapted to populations and conditions worldwide (Fried et al., 2016; Smarr and Keefer, 2011; Wang and Gorenstein, 2013).

Systematic reviews and meta-analyses have provided robust evidence of the BDI-II’s capacity for the accurate detection and assessment of depression (von Glischinski et al., 2019). Its strong psychometric properties have facilitated its adaptation as an e-mHealth tool in several regions worldwide [e.g., (Piers et al., 2025; Uchida et al., 2025)] and it remains suitable for adaptation in countries or populations where this version is not available.

In Mexico, the BDI-II is widely utilized across various health service settings, including primary care (Benuto et al., 2021), specialized services (Becerra-Gálvez et al., 2023), and monitoring treatment adherence (Gamiochipi-Arjona et al., 2021). Furthermore, it is a prominent instrument for evaluating depression risk and symptomatology in adolescents (Secundino-Guadarrama et al., 2021) and the general population, particularly during crisis periods such as the recent COVID-19 pandemic (Mestas et al., 2021). Nevertheless, a BDI-II version specifically adapted for e-health applications is currently absent in Mexico. Therefore, adapting the BDI-II for this modality would significantly enhance its utility and broaden its accessibility to diverse populations and settings.

In accordance with the best practices for the creation and adaptation of self-report scales in online environments, it is essential to conduct psychometric analyses to assess the impact of electronic adaptation on scale scores, as well as the performance of each item and the whole test (American Educational Research Association [AERA], American Psychological Association [APA], National Council on Measurement in Education [NCME], 2018; International Test commission, Association of Test Publishers, 2022).

There are two analysis frameworks in the literature to perform this task: Classical Test Theory (CTT) and Item Response Theory (IRT). The debate on the relative merits of each framework is extensive and beyond the scope of this article [for further information, see article such as those by Fan (1998)]. However, the literature highlights the advantages of using IRT for instruments for clinical and epidemiological use (Hays et al., 2000; Reise and Waller, 2009; Thomas, 2011; Thomas, 2019) since it makes it possible to obtain differentiated information for items and participants (such as performance, functionality across each trait level, difficulty of each item, trait level of participants and the amount of information on each item).

The IRT has been used to adapt the BDI-II to the general population (Fried et al., 2016), adults and older adults (Kim et al., 2002), adolescents (Arnarson et al., 2008; do Nascimento et al., 2023) and hospital populations (Almeida et al., 2023) and as an e-mHealth tool for patient evaluation in Australia (Williams et al., 2021) and South Korea (Park et al., 2020). It has also been used to analyze the structure of the instrument, which fluctuates between one-dimensional and two-dimensional positions (Wang and Gorenstein, 2013; Williams et al., 2021; Brouwer et al., 2013; Dere et al., 2015) and for determining how behavioral items (such as sexual behavior, eating behavior and sleeping patterns) tend to yield limited information in cultures with collectivist values, where there is strong pressure and social judgment on the expression of these behaviors (Wang and Gorenstein, 2013; Dere et al., 2015). Conversely, in individualistic cultures, limited information tends to be available on items reflecting affective symptomatology (Dere et al., 2015; Byrne et al., 2007).

To the best of our knowledge, in Mexico, the BDI-II has been only analyzed using CTT, with studies that have provided evidence of validity and accuracy in various populations for the paper-and-pencil version of the instrument. These studies have found a wide diversity of first-order structures in the general population, particularly two-factor structures, since they coincide with most structures found internationally, albeit with differences in the amount and order of the items comprising them depending on the region being analyzed, in other words, in the north (Estrada Aranda et al., 2015) or southeast (Rosas-Santiago et al., 2020) of the country (Table 1). Using these studies as a basis makes it possible to undertake more specific analyses from the IRT framework that provide new evidence and information on BDI-II items and their composition.

**Table 1:** Factor models for the BDI-II in Mexico.

Region	No. of factors	Name of factors	Items in factors	Cronbach’s Alpha
Southeast (Rosas-Santiago et al., 2020)	2	1. Cognitive-affective dimension	1, 2, 3, 4, 5, 6, 7, 8, 9, 10, 12, 13, 14	0.89
		2. Somato-vegetative dimension	11, 15, 16, 17, 18, 19, 20, 21	0.83
North (Estrada Aranda et al., 2015)	2	1. Cognitive-affective dimension	1, 2, 3, 5, 6, 7, 8, 9, 10, 11, 13, 17	0.85
		2. Somato-vegetative dimension	4, 12, 15, 16, 18, 20, 21	0.78

The objective of this study was therefore to provide psychometric evidence for the adaptation of the BDI-II as an e-mHealth tool, based on the analysis within the IRT framework making it possible to provide evidence on the dimensionality of the instrument and the performance of the items based on their interpretation, cultural and sociodemographic sensitivity, for measuring the degree of depression in online settings for the general population in Mexico.

## 2 Method

### 2.1 Study design

A retrospective, predictive and secondary analysis was conducted of the records of individuals over 18 using the online self-diagnosis platform of Centros de Integración Juvenil (Youth Integration Centers, Spanish acronym CIJ) to screen for depressive symptoms between February 2021 and June 2022. Information was collected through an online questionnaire available 24/7 on the http://www.cij.gob.mx/autodiagnostico/index.asp website.

### 2.2 Participants

The sample was obtained through a non-probability convenience sampling process, comprising participants of legal age (18 years or older) who submitted their responses between February 8, 2021, and June 16, 2022. Due to the nature of the self-screening website (absence of formal registration, identification data, or other identifying information), no further controls were implemented. Exclusion criteria included belonging to the LGBTIQ+ community or being indigenous. This decision was made because evidence regarding the incidence of depression in these populations reveals distinct characteristics, such as high prevalence, specific risk factors (e.g., discrimination and stigma), and complex conditions like intersectionality (Cai et al., 2024; Meldrum et al., 2023). These factors necessitate a separate study design and a tailored process of adaptation and validation of the BDI-II for these populations, which would be compromised if aggregated with the main sample.

### 2.3 Instruments

An electronic version of the Beck Depression Inventory-II (Beck et al., 1996) adapted for mexican population was used. This version comprises twenty-one items designed to measure the cognitive or emotional processes associated with depressive symptoms in the past 2 weeks. It has evidence of accuracy and validity in several countries (Arnarson et al., 2008; do Nascimento et al., 2023) and specific populations (Almeida et al., 2023; Eser and Asku, 2021; Kühner et al., 2007), as well as high accuracy with an estimated internal consistency of over 0.89 and test–retest reliability of 0.75 (Eser and Asku, 2021; Erford et al., 2016). In addition, it has appropriate cut-off points (von Glischinski et al., 2019), enabling it to be used as a screening scale for depressive disorder.

To obtain sociodemographic information, participants answered a brief questionnaire on their age, sex (male, female, or unspecified), and state of residence. Lastly, the response system identified each record with the date and time of completion of the questionnaire.

### 2.4 Information collection procedures

Data were collected from the platform,^1^ where users can answer questions about their depressive symptoms (BDI-II) and fill out a brief sociodemographic information sheet. Once the questionnaire has been completed, the platform provides automated feedback on the level of depression obtained, giving users a range of options where they can seek care.

### 2.5 Ethical procedures

The research was conducted in keeping with the recommendations of the International Ethical Guidelines for Health-related Research Involving Humans (International Union of Psychological Science, 2008) and the Ethical Principles of Psychologists. The Code of Conduct (American Psychological Association, 2016) was followed for the preparation and presentation of informed consent, privacy notices, personal data management policy, privacy risks and the ways these risks are minimized.

Before completing the online self-diagnosis questionnaire, users must read and approve the privacy and use of personal data notices specifying that all the data provided can be used for research and publication purposes. The platform does not request any electronic identification data from users (such as name, address, email, location or IP address) and each user’s records and responses are anonymous and confidential. The protocol was submitted for evaluation by the institutional scientific research committee of CIJ (number: 22-03), which evaluated the methodological relevance and adherence to ethical criteria.

### 2.6 Data analysis

Frequencies and percentages of the sociodemographic variables and BDI-II scores were obtained. The fit of the BDI-II to Samejima’s graded response model (Samejima, 1969) was tested, and the fit of four different factor structures was subsequently analyzed:

Models with a two first-order factor structure:

Southeastern model, proposing a first cognitive-affective factor (items: 1, 2, 3, 4, 5, 6, 7, 8, 9, 10, 12, 13, 14) and a second one called somatic-vegetative (items: 11, 15, 16, 17, 18, 19, 20, 21).
The northern model assumes two first-order factors: a cognitive-affective one (items 1, 2, 3, 5, 6, 7, 8, 9, 10, 11, 13, 17) and a second somatic-vegetative one (items 4, 12, 15, 16, 18, 20, 21).

Bi-factor or hierarchical first and second order factor models (Brouwer et al., 2013; Toland et al., 2017):

The southeastern model comprised the two first-order factors, both loading onto a second-order dimension of ‘depression severity’ encompassing all items.
Similarly, the northern model also featured its two first-order factors, with all items loaded onto a second-order ‘depression severity’ factor.

The four models were analyzed by undertaking IRT confirmatory analyses (Toland et al., 2017), in which structures were defined through the mirt.model function and their parameters estimated. In the case of bi-factor models, items were assigned to a latent variable, identifying each one with its belonging factor, and subsequently analyzed with the bfactor function. These analyses were undertaken with the mirt library (Chalmers, 2012).

To assess the models, their general adequacy was first tested through the CFI (>0.90), TLI (>0.90) and RMSEA (<0.08) indices in their C2 adequacy for items with an ordinal response format (Cai and Monroe, 2014). Their performance was subsequently compared by evaluating two parsimony criteria: the Bayesian Information Criterion (BIC) and the Akaike Information Criterion (AIC) which, based on the recommendations of (Anderson, 2007), make it possible to evaluate the adequacy of a model based on the reduction of the values of these indices. Anderson himself declared that a difference of nine or more between models is considered solid evidence of the fact that one model is more suitable than another. The characteristics of the items and information functions were obtained from the model with the best fit. Within a bifactor model, item information enables the evaluation of each item’s fit to the model’s assumptions, considering both first-order factors and the higher-order dimension. Item information functions, which are graphical representations, depict the amount of information yielded about the underlying trait (the depression severity) by each item. A larger area under the curve signifies greater item information.

To complete the analyses, precision estimates were obtained for the model with the best fit through Cronbach’s Alpha and McDonald’s Omega coefficients, using the psych library (Revelle, 2017). All analyses were performed using the R programming language version 4.3.2 (R Core Team, 2023) in the R Studio integrated development environment, version 2022.12.0 (Posit Team, 2023).

## 3 Results

**Figure 1:**
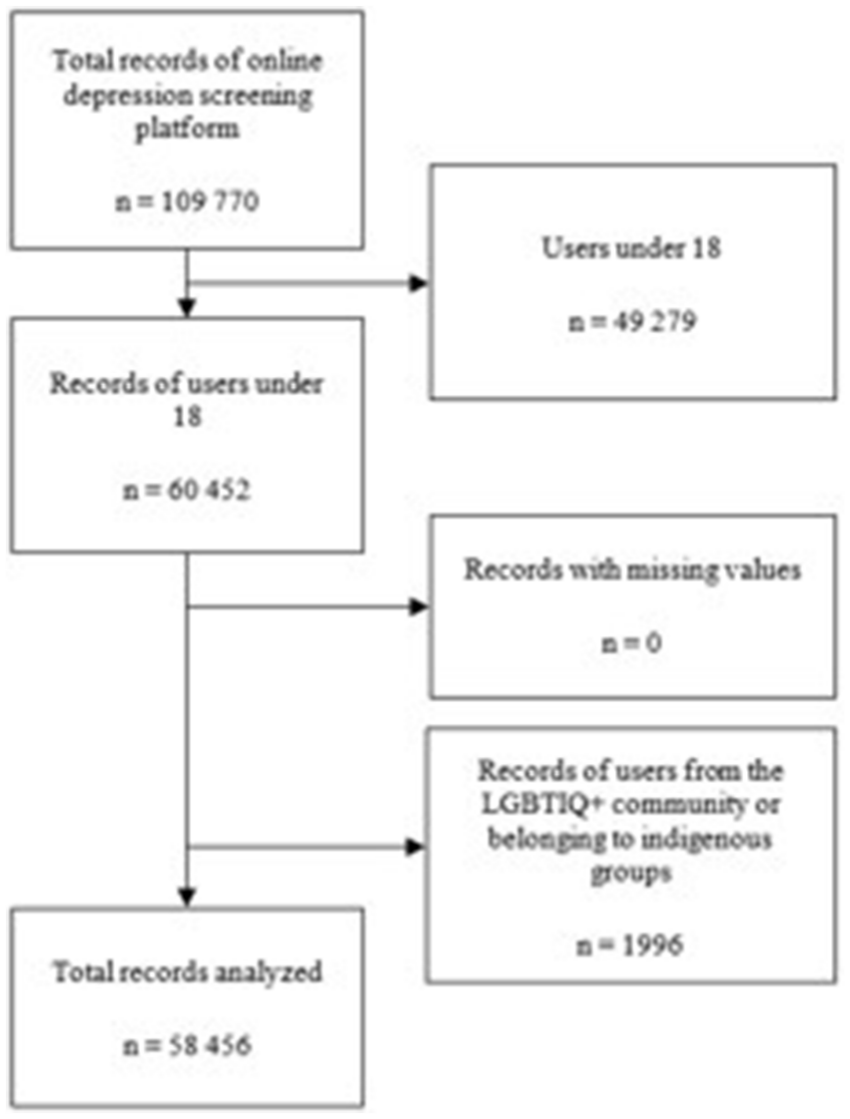
Flowchart of records analyzed. Description: Refers to the filtering of data based on inclusion, exclusion and elimination criteria.

Of the total number of participants using the platform, 49,279 (44.89%) were eliminated for being minors, and 1,996 records (1.8%) were eliminated for failing to meet the inclusion and exclusion criteria, yielding a total of 58,456 records available for analysis (See Figure 1).

### 3.1 Sociodemographic data

The sample consisted mainly of adults (*M* = 27.86 years, SD = 9.62), 41,450 (70.91%) of whom were women and 17,006 (29.09%) men (according to their sex assigned at birth) and aged between 18 and 80 (*M* = 27.86, SD = 9.62). Mean depression was 30.95 (SD = 13.51, with a range of 21 to 63) while the proportion of cases at each level of severity was 53.3% severe, 28.7% moderate, 7.97% medium and 6.97% minimal. The highest proportion of responses was from residents of the Mexico City metropolitan area (22.14% from Mexico City and 9.29% from the State of Mexico).

### 3.2 Comparison of factor structures

The four aforementioned factor structures were evaluated through confirmatory IRT models. The first-order two-factor structures showed a poor fit, while the hierarchical two-factor structure based on the Southeastern model of Mexico failed to show a solution after 500 iterations, so its convergence was not considered. Finally, the northern hierarchical bifactor model showed a good fit in the three indices considered (CFI_C2_ = 0.984, TLI_C2_ = 0.979, and RMSEA_C2_ = 0.040).

The fit measures for all estimated models can be seen in Table 2, while the fit of the items for the best model (the northern hierarchical bifactor model) is shown in Table 3.

**Table 2:** Global fit and comparative measures of IRT confirmatory GRM models for the BDI-II.

Models	*C2 (gl)*	*CFI_C2_*	*TLI_C2_*	*RMSEA_C2_*	*AIC*	*BIC*
First order Southeast	85281.99 (148)	0.889	0.874	0.099	2,832,633	2,833,378
First order North	97647.09 (149)	0.873	0.857	0.105	2,868,686	2,869,422
Second order Southeast	8924.78 (127)	0.988	0.984	0.034	2,729,154	2,730,088
Second order North	**12125.29 (128)**	**0.984**	**0.979**	**0.040**	**2,738,252**	**2,739,176**

The Southeast second order did not converge after 500 iterations, as a result of which its fit estimates are unreliable. Bold values in the table represent the best-fitting GRM model for the BDI-II.

**Table 3:** Individual fit and BDI-II item betas in the one-dimensional model of the North.

Item	Chi^2^	*gl*	*p*	*RMSEA*	*aG*	as1	as2
R1	510.872	153	<0.001	0.006	2.13	1.16	0
R2	297.566	159	<0.001	0.004	1.89	0.69	0
R3	413.294	159	<0.001	0.005	1.99	0.66	0
R4	283.620	156	<0.001	0.004	1.7	0.49	0
R5	1537.091	157	<0.001	0.012	1.83	0.43	0
R6	1765.004	167	<0.001	0.013	1.75	0.43	0
R7	2454.099	160	<0.001	0.016	1.4	0.4	0
R8	518.819	156	<0.001	0.006	1.94	0.13	0
R9	356.948	158	<0.001	0.005	1.92	0.12	0
R19	370.760	157	<0.001	0.005	1.07	−0.05	0
R20	1296.982	151	<0.001	0.011	1.09	−0.25	0
R21	677.701	168	<0.001	0.007	1.67	−0.37	0
R10	3228.601	168	<0.001	0.018	1.85	0	0.36
R11	1767.213	168	<0.001	0.013	1.87	0	0.48
R12	1190.218	156	<0.001	0.011	2.29	0	0
R13	966.296	158	<0.001	0.009	2.56	0	1.76
R14	385.622	155	<0.001	0.005	1.21	0	0.5
R15	598.341	146	<0.001	0.007	1.41	0	0.41
R16	674.427	165	<0.001	0.007	1.86	0	0
R17	1201.119	163	<0.001	0.010	2.37	0	1.6
R18	462.617	163	<0.001	0.006	0.9	0	0.37

aG = General severity factor, as1 = cognitive-affective factor, as2 = somatic-behavioral factor. RMSEA levels < 0.08 demonstrate adequate fit.

**Figure 2:**
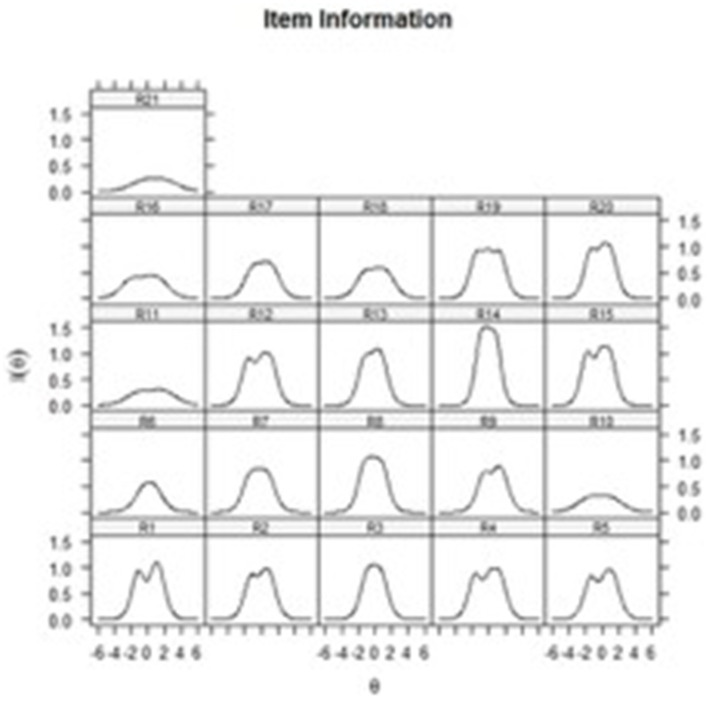
Information functions of the BDI-II items. Description: Graphs showing how much information on depression each item recovers across all the trait values. Items with higher density graphs have more information on depression than items with less dense, flattened graphs.

After confirming that a hierarchical structure with a higher-order dimension provides the best fit, it was decided to continue the analyses using this structure. The first-order dimensions were restricted to zero and the information functions of the items were obtained to evaluate the capacity of each one to provide the greatest amount of data on the higher-order dimension (depression severity level). These functions can be seen in Figure 2, in which it is striking that items 10, 11, 16 and 21 contribute less information than the other items.

**Figure 3:**
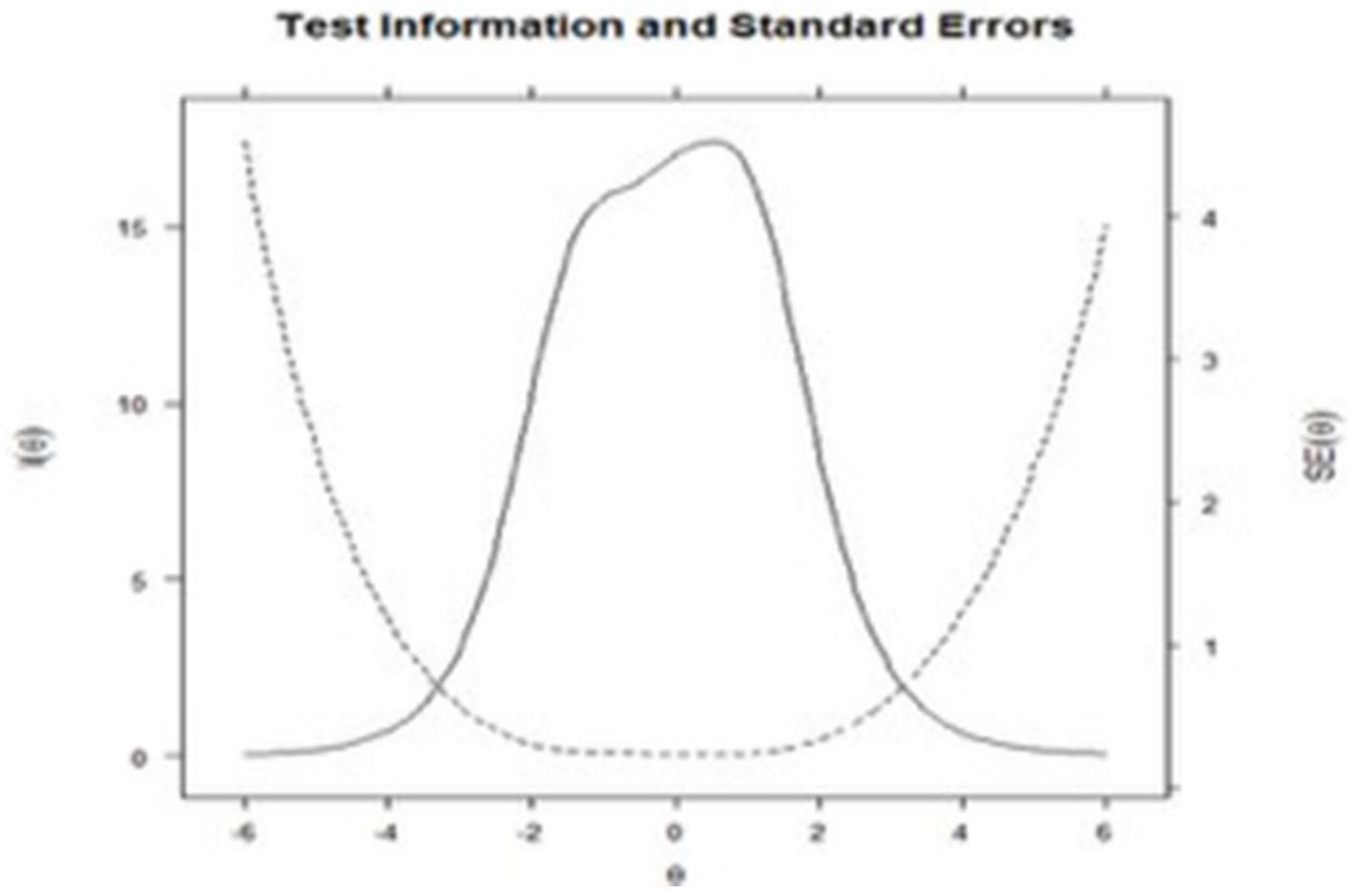
Global information function and standard error of the BDI-II. Description: Shows the amount of information on the latent trait available from the entire set of items and is compared with a dotted line referring to measurement error.

Evidence of accuracy for the scale was subsequently obtained. Good performance was observed in the test information graph in contrast to standard error (Figure 3), showing that the BDI-II accurately evaluates medium and moderate levels of the depression trait. This is also confirmed by the point estimates throughout the test, evaluated through the Cronbach’s Alpha index = 0.93 and MacDonald’s Omega = 0.93.

### 3.3 Depression severity

**Figure 4:**
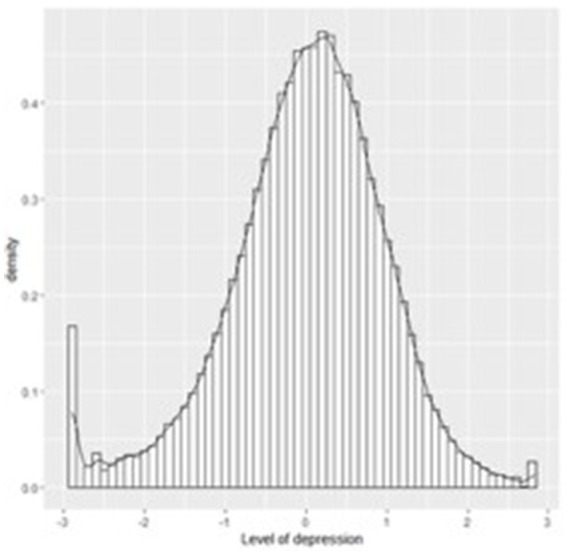
Severity of the latent trait in the sample based on BDI-II scores. Description: Shows the distribution of the latent trait values along a continuum, enabling the most prevalent depression values to be identified in the sample.

Finally, the latent trait score (theta) was estimated. This score refers to the level of severity of the phenomenon, in this case depression, in the study population (Castro et al., 2010; de Francisco et al., 2015). The data show that the depression values with the highest probability of occurrence are medium and moderate (*M* = −0.00018, range − 2.909–2.836). The complete estimated trait values can be seen in Figure 4.

## 4 Discussion

For this study, a psychometric evaluation of the BDI-II was conducted using the IRT framework, to provide evidence for its use as an e-mHealth tool in the Mexican population. In general, results support the use of the instrument for the identification of cognitive-affective and somatic-behavioral symptoms of depression, as well as for inferring/interpreting the degree of severity of this condition in users evaluated with the BDI-II.

Traditionally, the interpretation of BDI-II scores is linked to its factor structure, where there are two main positions: the structure of two first-order factors (cognitive and somatic factors) or the hierarchical two-factor structure that adds a second-order (or higher-order) dimension to the first proposal to measure the degree of severity of depression symptoms (Wang and Gorenstein, 2013; Byrne et al., 2007). Thus, when first-order structures are tested for BDI-II, it is only possible to draw inferences from the total scores of each factor (somatic-behavioral or cognitive-affective expressions) rather than from a global score, which constitutes a significant limitation when drawing conclusions about the general level of depression of those answering the instrument.

The hierarchical two-factor alternative implemented in this study overcomes this limitation by allowing both levels of analysis: the first order corresponding to the behavioral-somatic or cognitive-affective expressions of depression and the higher order making it possible to infer the overall severity of depression (Williams et al., 2021; Brouwer et al., 2013; Dere et al., 2015).

The study also provides evidence of the structure of the BDI-II found in the northern region of Mexico (Estrada Aranda et al., 2015), enhancing its findings with the higher dimension already mentioned, and suggesting that this structure could be the most useful one for drawing inferences from the BDI-II among the Mexican population. Although the study by (Estrada Aranda et al., 2015) analyzed the adequacy of a two-factor structure, their analysis did not involve comparing the fit of different models or a hierarchical organization of factors, since it only explored the adequacy of a first-order two-factor model. The current study proves that the organization of two factors underlies a higher order factor and describes the model as bifactor due to the name of the hierarchical model proposed in the literature (Toland et al., 2017).

At the same time, due to the advantages of IRT as an analytical framework (Hays et al., 2000; Reise and Waller, 2009; Thomas, 2011; Thomas, 2019), the performance of the items was evaluated based on the information they provide about the condition. These analyses showed that items 10 (“Crying”), 11 (“Agitation”), 16 (“Changes in sleeping habits”) and 21 (“Loss of interest in sex”) contribute the least information across all depression values, which translates into items that are not useful for its identification among the mexican population. The limited information provided could be explained by the influence exerted by culture, as well as the non-clinical conditions of the sample with which we worked. Previous studies have suggested that cultures with collectivist values, such as that of Mexico (Triandis and Gelfand, 2012), exert negative pressure on the expression of behavioral symptoms related to sexuality, preventing people from freely reporting changes in themselves (Wang and Gorenstein, 2013; Dere et al., 2015; Byrne et al., 2007). In regard to items 10, 11 and 16, it has been suggested that since the BDI-II was created to identify symptomatic expressions in clinical populations, it fails to capture response variations in certain items that refer to more obvious expressions of depression in patients such as crying, noticeable agitation and changes in sleeping habits (Byrne et al., 2007; Dawes et al., 2010), which explains their low contribution of information.

This is borne out by a recent study using machine learning to determine that items 11 (“Agitation”), 19 (“Difficulty concentrating”), 18 (“Changes in Appetite”), 16 (Changes in sleeping habits”) and 20 (“Tiredness or fatigue”) in the BDI-II, predict mental health treatment-seeking behaviors in a population assumed to be clinical (Sánchez-Domínguez et al., n.d.), showing that the variations in these items are more useful and functional for this type of population.

Regarding the accuracy estimates of the instrument, the indices calculated from the CTT (Cronbach’s Alpha and MacDonald’s Omega) showed optimal performance, suggesting a stable evaluation of depression by the BDI-II and its factors, while the estimation of the IRT (information function as opposed to standard error) showed that the highest levels of accuracy were found for those with medium and moderate levels of depression. These results contribute to the research and systematic reviews of the BDI-II, describing it as an accurate scale (Eser and Asku, 2021; Erford et al., 2016).

The psychometric evaluation of the instrument, for its use as an e-mHealth tool, supports its potential implementation as a self-assessment measure, thereby expanding access to health services, particularly in the public sector. This would facilitate the early identification of depression and provide individuals with crucial insights into their mental health status, empowering them to make informed decisions regarding treatment and care. Furthermore, the identification of items yielding less information presents at least two distinct applications. At a clinical level, if prioritizing rapid administration of the instrument is desired, these items could be considered for initial elimination, resulting in a shortened version that would likely retain a robust capacity for detecting depression while omitting only minimally informative items. Additionally, from a cultural analysis perspective, the low information content of these items may indicate that symptoms such as crying, agitation, changes in sleep patterns, and diminished sexual interest are not significantly discriminating factors for identifying depression within the general, non-clinical mexican population. This warrants consideration in diagnostic manuals and guidelines for depression screening within the country. However, given the nature of the sample, this observation remains speculative and requires further empirical validation.

Finally, the level of severity observed in the sample is medium to moderate, despite the fact that the sample is from the general population. It is possible that this level of severity can be explained by the fact that the responses received in the online questionnaire come from people who are seeking to confirm a feeling of discomfort in their mental health. Therefore, although they are from the general population, they tend to present higher levels of the trait than healthy people, due to the self-perceived discomfort prior to evaluation.

### 4.1 Limitations of the study

This study is not without its limitations. Although sample size was optimal for conducting IRT analyses and making accurate estimates of the instrument, the data cannot be considered representative of the general population due to the lack of a randomly obtained representative sample. Future studies should therefore conduct sampling through randomized, representative procedures, at least in each region, to replicate the analyses conducted.

Likewise, although the study focused on providing evidence of validity and accuracy for the use of the BDI-II as an online self-diagnosis tool, evidence related to the impartiality or invariance of the instrument was not examined. These analyses should be performed in new studies to obtain evidence making it possible to compare scores between users with different characteristics.

Additional studies should also be undertaken to obtain other evidence of validity, such as that referring to the relationship with other variables (before called validity convergent, divergent and/or concurrent), negative consequences of using the test or the process of answering the scale. These analyses should be conducted on various groups of users, such as those who use psychoactive substances or those with other medical conditions to improve evidence of the usefulness of the instrument for the early identification of this condition in various population groups.

### 4.2 Conclusion

In conclusion, evidence was obtained on the use of the BDI-II to measure depressive symptoms through an online self-diagnosis platform, so that it can be used as an e-mhealth tool. The results support the use of the instrument as an online identification tool for depression, and its total score can be interpreted as the degree of severity of the condition.

Evidence was also obtained of a two-factor hierarchical structure, contributing to the theoretical debate on the internal structure of the scale (evidence of validity referring to the internal structure). The fit of the items with the graduated response logic was verified (evidence of validity of the response process). Items contributing limited information were identified, supporting findings on the sensitivity of behavioral items to cultural and clinical variations (evidence of validity referring to content), as well as those useful for the evaluation of depression. Finally the precision of the questionnaire was analyzed, yielding high estimates (evidence of accuracy).

These results support the use of the BDI-II as an online self-diagnosis instrument for depression, whereby valid inferences can be made about the degree of severity of this condition based on the total score.

## Data Availability

The raw data supporting the conclusions of this article will be made available by the authors, without undue reservation.

## Footnotes

^1^ http://www.cij.gob.mx/autodiagnostico/frmdepresion.asp
